# Knowledge and awareness of the Saudi general public toward epistaxis: a cross-sectional study

**DOI:** 10.3389/fpubh.2024.1269559

**Published:** 2024-05-27

**Authors:** Abdullah M. Assiri

**Affiliations:** Department of Surgery, College of Medicine, Najran University, Najran, Saudi Arabia

**Keywords:** epistaxis, knowledge, management, first aid, Saudi Arabia

## Abstract

**Background:**

Epistaxis is one of the most common ear, nose, and throat (ENT) emergencies that present to the emergency or primary care centers.

**Study aim:**

This study aimed to assess the knowledge of the Saudi general public toward epistaxis.

**Methods:**

This study adopted a cross-sectional analytical study design. The questionnaire link was distributed using social media channels. The participants were adult Saudi nationals that live in Saudi Arabia. The data was collected using a self-administered questionnaire that assessed knowledge related to epistaxis. The knowledge score was calculated using the 10 knowledge evaluation questions. Each correct response was assigned a value of “one.” The scores ranged from “zero” to “ten,” with higher scores signifying greater knowledge. A percentage score was computed, and the participants’ knowledge was classified as poor (% score: ≤50%), moderate (% score: 51 to 70%), and good (% score: 71 to 100%). Statistical Package for Social Sciences (SPSS) version 26 was used for statistical analysis.

**Results:**

The study included 452 participants of whom 70.1% were females. Married individuals comprised 60.8% of the sample. The prevalence of self-reported epistaxis was 43.6% in the last 6 months. Among the participants, 42.9%, had “Poor” knowledge score, followed by 39.6% who had “moderate” score, and 17.5% had “Good” score. These results show that most participants had poor to moderate knowledge, with a minority demonstrating a good level of knowledge. All demographic variables have significantly influenced the adequacy of knowledge about epistaxis. Furthermore, participants who believed that the general public has insufficient knowledge on epistaxis had a significantly lower knowledge score (*p* = 0.001).

**Conclusion:**

The present study found a non-satisfactory, low-to-moderate knowledge level of the Saudi general public toward epistaxis. We propose emphasizing public knowledge and education about first aid for epistaxis because proper first aid can minimize significant complications when done properly.

## Introduction

One of the most frequent emergencies in Ear, Nose, and Throat (ENT) and Accident and Emergency (A&E) departments is believed to be epistaxis. Epistaxis can range in severity from moderate to severe, life-threatening bleeding. It is a challenge to otolaryngologists since it makes patients and medical staff nervous and lasts more than an hour in the majority of instances (1). While it is uncommon in new-borns and children under 2 years of age, it is a frequent disorder in children and young adults (2). Between the ages of 3 and 8 years, it frequently happens, and as people become older, the prevalence drops (3). According to reports, 10 to 60% of people have epistaxis (4), and 50% of all adults have had it at some point in their childhoods. According to reports, 7 to 14% of the general population are hospitalized for epistaxis-related problems each year (5, 6). Systemic variables, such as blood disorders, anticoagulant usage, and coagulopathy, as well as local ones, such as trauma, nasal septum perforation, nasal hypersensitivity, infections in the upper respiratory system, and the entry of foreign substances into the nasal cavity, can all result in nasal hemorrhage (7). First aid is the emergency therapy for epistaxis, which is crucial to reduce suffering and stop the situation from getting worse. The bleeding can be stopped with pressure or it may cease on its own (8). However, some epistaxis patients necessitate hospitalization and pharmacological intervention (9).

Since most episodes are brief, those who are affected rarely seek medical attention. Rarely, there may also be severe epistaxis (10). Certain epistaxis cases may be treated conservatively using anterior nasal packing, which applies pressure directly to the areas where bleeding occurs.

Anatomically, epistaxis typically originates from the Little’s area, also known as the Kisselbach’s plexus (11, 12). Most of the nose bleeds have some cause, including but not limited to nose picking, trauma, allergies, dry air, infections, nasal tumors and hypertension. Nose bleeds are typically benign, and self-limiting, and it may be treated at home with the proper first aid. But to achieve it, awareness and sufficient knowledge are needed (13).

Previous studies on epistaxis have revealed that there is a knowledge gap related, and an urgent need for early intervention and timely administration of first-aid measures to prevent complications. A recent study in Makkah, Saudi Arabia, revealed that the overall knowledge of proper epistaxis first aid was poor, with only 437.1% participants demonstrating good knowledge (14). Similarly, another study revealed significant gaps in the knowledge and practices of first aid for epistaxis among the general population in the Jazan region, Saudi Arabia (15). In another study in Riyadh, Saudi Arabia, the general public’s practices and knowledge were found to be adequate. In all, 81% of the participants were able to properly identify the first step of epistaxis first-aid management (16). A research in Taif, Saudi Arabia, examined parents’ knowledge of first-aid treatment for epistaxis in children. According to the survey, most Saudi parents had a modest degree of understanding of first-aid treatment for epistaxis (17). In Nigeria, a 7-year cross-sectional prospective study was conducted, a questionnaire was provided to consenting parents of children who arrived to the tertiary care hospital with epistaxis between January 2015 and December 2021. The study found that a significant percentage of parents were unaware of epistaxis, as well as its aetiological and risk factors (18). Hence, campaigns to raise awareness among the general public and educational programs require urgent attention.

Therefore, this investigation was conducted to assess the knowledge of Saudi population toward epistaxis. In contrast to other researches that concentrated on the prevalence of epistaxis in general populations, the current study sought to assess the knowledge of the general public (Saudis) toward epistaxis in Saudi Arabia. The scientific impact of this study lies in its potential to address knowledge gaps, improve self-management, promote early intervention, reduce unnecessary healthcare utilization, and inform healthcare policies and guidelines. By identifying areas of limited understanding or misconceptions regarding epistaxis, the study can guide the development of targeted educational campaigns and interventions to address these knowledge gaps. Increasing public awareness and knowledge about the causes, risk factors, preventive measures, and appropriate management techniques can empower individuals to take appropriate actions during nosebleed episodes and potentially reduce their severity.

## Methodology

### Study design and data collection

This study utilized an analytical questionnaire-based cross-sectional design. A self-administered questionnaire was used for data collection. Designing the questionnaire was based on a review of existing literature (19–26). To ensure that the research instrument was a good fit for the characteristics of the subjects, it went through a rigorous process of customization. The first part of the self-administered survey collected basic demographic information, while the second part tested participants’ knowledge and asked about any previous experiences with epistaxis. The second part contained 10 questions to assess the knowledge of the participants.

### Calculating the knowledge score

We computed the knowledge score using the 10 question items pertaining to assessment of knowledge. Each response was assigned a value of “one” for correct answer and “zero” for wrong answers. The overall knowledge score was determined by summing the appropriate accurate answers. Consequently, the scores ranged from “zero” to “ten,” with higher scores signifying greater knowledge. A percentage score was computed, and the participants’ knowledge was classified as poor (% score: ≤50%), moderate (% score: 51 to 70%), and good (% score: 71 to 100%).

### Study duration

The data was collected using a self-administered form during the period from June 2022 to July 2022.

### Study population

The study included data on adult Saudi individuals, who are currently living in Saudi Arabia, and those who were willing to participate in the survey.

#### Validity and reliability of study tools

Experts from the department of surgery, department of family and community medicine, and department of epidemiology, Najran University, reviewed and analyzed the questionnaire’s preliminary form to check its content and face validity. A neutral professional translator first translated the questionnaire into Arabic, and then a second professional translator back into English to ensure consistency. The final questionnaire was in the Arabic language and distributed online through a questionnaire link on social media platforms. Twenty people participated in a pilot study to test the reliability and validity of the questionnaire. Results from the pilot study’s subjects were added to the final count. As a further note, Cronbach’s alpha factor was calculated for each question on the questionnaire to determine its reliability, and it was found to be 0.81, indicating a high degree of internal consistency.

#### Technique for sampling, sample size, and distribution of study instrument

To gather the required sample, we used a convenient sampling procedure. According to the World Population Review^1^, the overall population of Saudi Arabia is estimated to be 36,408,820 in 2022. Among them the Saudis account for two thirds of the population and the remaining one third are expatriates. Thus, the Saudi nationals’ population is estimated to be 22,426,564. Using the Raosoft sample size calculator^2^, a minimum sample size of 385 was computed using the 95% confidence level, a margin of error of 5%, and a sample percentage of 50%. We distributed the questionnaire link using social media channels such as Twitter, LinkedIn, Facebook, and WhatsApp status. This method facilitated the inclusion of a wide range of individuals from various geographical regions, age groups, and cultural backgrounds. The objectives of the survey and the informed consent were clearly stated at the beginning of an online questionnaire. After reviewing the consent form and study purpose, participants had the option to proceed with the study by selecting the “agree icon” or decline participation by selecting the “disagree icon.” This ensured that participation was entirely voluntary. Disagreeing individuals were directed to decline the participation section and exit the survey.

### Data management and statistical analysis

The Statistical Package for Social Sciences (SPSS) version 26 was used for data management and analysis. The sociodemographic data was presented as frequencies and percentage. The Chi-square test was employed to determine the statistically significant differences between the categorical variables and the knowledge score and independent *t*-test followed by one way ANOVA (*post Hoc* Tukey) was used to determine the association and significance in mean knowledge score, where a *p* value ≤0.05 was considered significant.

### Ethical considerations

The study was conducted in accordance with the Declaration of Helsinki and approved by the Institutional Review Board of Najran University (NU/IRB/2021/12/3). All participants were given an informed consent form, and the questionnaire was designed to protect the privacy of all responses. Finally, participants were reassured that their anonymity would be protected when the study’s findings were made public.

## Results

The study included 452 participants, of whom 70.1% were females. Over half the participants were 21 to 30 years old (52.2%). The majority (60.8%) of the participants were married, had a university degree (73%), and believed that the general public does not have enough knowledge of epistaxis (86.1%). The prevalence of self-reported epistaxis was found to be 43.6% (Table 1).

**Table 1:** Sociodemographic characters of participants (*n* = 452).

Demographic variables		Demographic characteristics (*N*)	Percentage
Gender	Male	135	29.9
	Female	317	70.1
Age	Less than 20 years	41	9.1
	21–30 years	236	52.2
	31–40 years	102	22.6
	41–50 years	33	7.3
	51 years and above	40	8.8
Marital status	Unmarried	159	35.2
	Married	275	60.8
	Divorced/separated	18	4
Education level	Illiterate	22	4.9
	Intermediate education	7	1.5
	Secondary school	93	20.6
	University graduate	330	73
In my opinion, the general public has insufficient knowledge on epistaxis	No	20	4.4	Unsure	43	9.5	Yes	389	86.1
	History of epistaxis in the last 6 months	No	255	56.4	Yes	197	43.6

**Figure 1:**
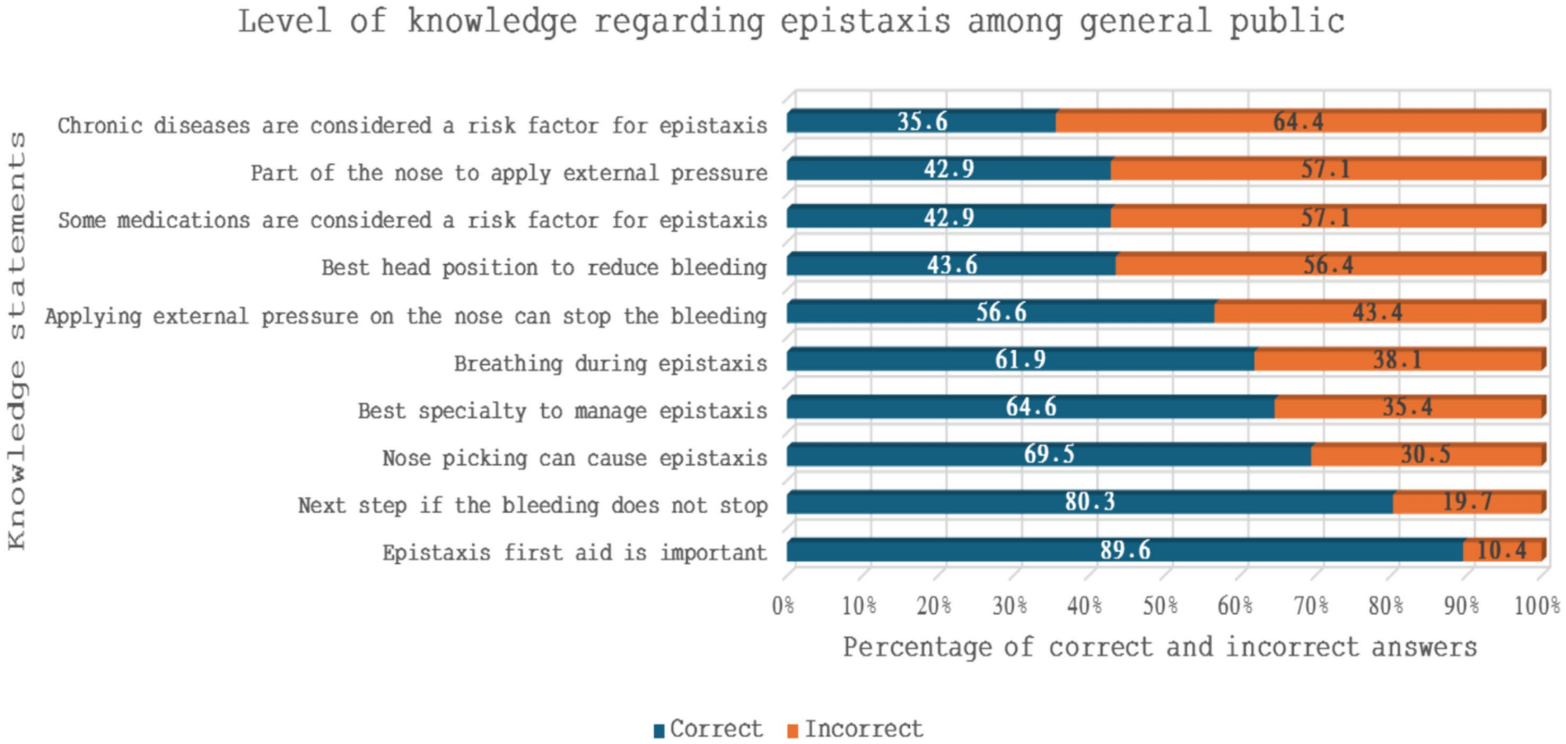
Level of knowledge regarding epistaxis among general public.

The participants’ level of knowledge was assessed using 10 statements (Figure 1). The highest percentage of correct answers were observed for “Epistaxis first aid is important (89.6%),” “what is the next step if bleeding does not stop (80.3%)” and “nose picking can cause epistaxis (69.5%).” Conversely, high percentage of incorrect answers were recorded for “chronic diseases are considered a risk factor for epistaxis (64.4%),” “part of the nose to apply external pressure (57.1%),” and “some medications are considered a risk factor for epistaxis (57.1%)” (Figure 1).

**Figure 2:**
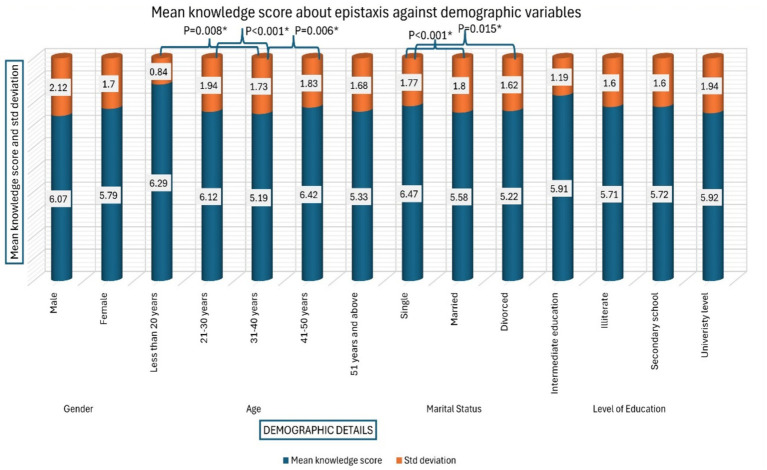
Mean knowledge score about epistaxis against demographic details.

Figure 2 depicts the mean knowledge score of participants regarding epistaxis against demographic details. An independent *t*-test followed by one way ANOVA (*post Hoc* Tukey) was performed to find out the significance. The mean knowledge score among males (6.07) was non-significantly higher than females (5.79). Whereas, participants aged less than 20 years demonstrated significantly better mean score (6.29) compared to those aged 31–40 years (score-5.19, *p* = 0.008). Similarly, participants aged 21–30 years have shown significantly (*p* < 0.001) better knowledge score (6.12) compared to 31–40 years old participants. Likewise, participants aged 41–50 years have demonstrated significantly (*p* = 0.006) better knowledge score (6.42) compared 31–40 years old participants. Unmarried participants had significantly (*p* < 0.001) better mean score (6.47) compared to married (5.58), and divorced participants (score-5.22, *p* = 0.015). We did not observe any significant impact of level of education on mean knowledge score.

**Figure 3:**
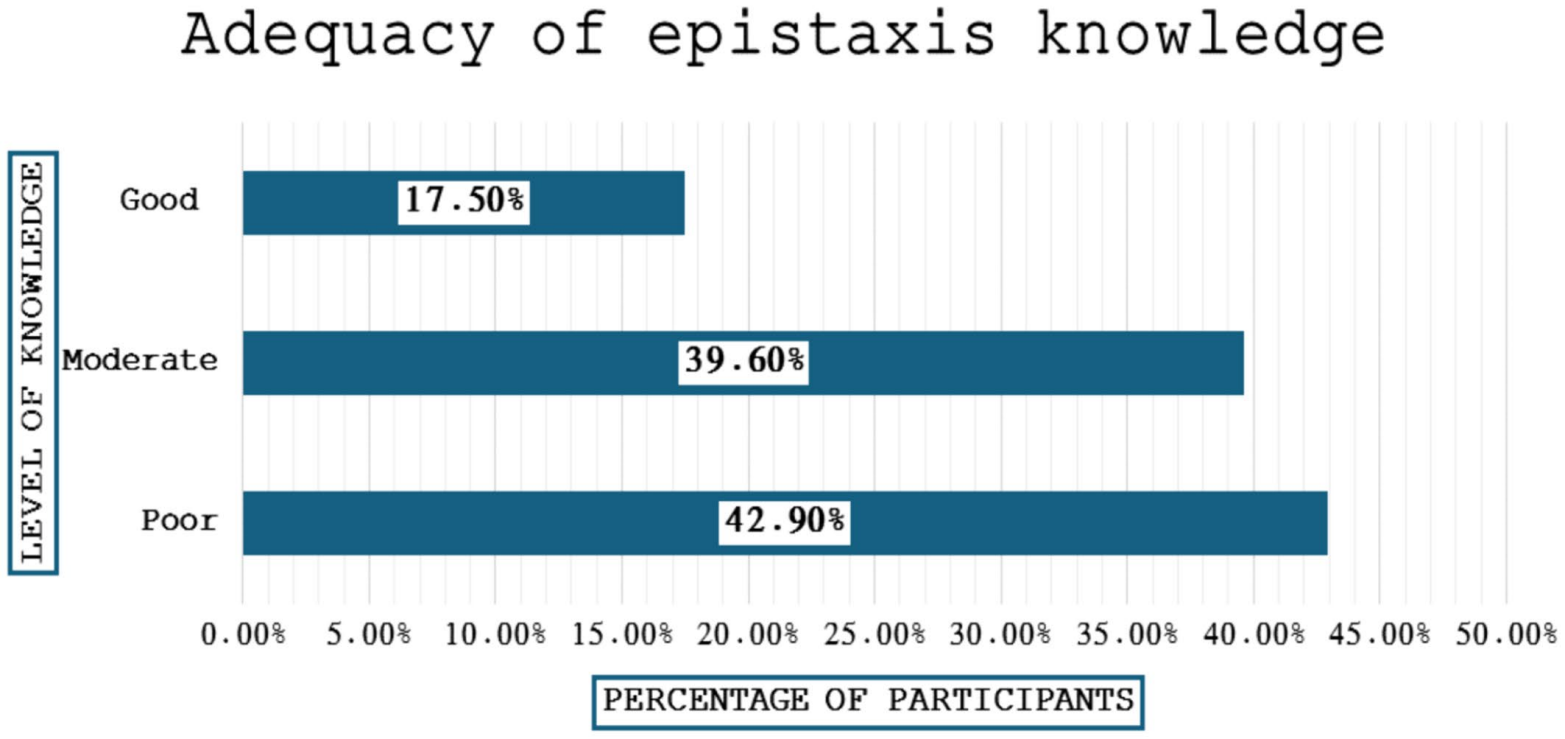
Adequacy of knowledge regarding epistaxis.

Figure 3 shows the adequacy of knowledge regarding epistaxis. The participants with poor, moderate, and good knowledge were 42.9, 39.6 and 17.5%, respectively.

Table 2 depicts the association between demographic variables and level of knowledge. A statistically significant association was observed among all demographic variables. The male participants with good knowledge (24.4%) were significantly (*p* = 0.038) higher compared to females (14.5%). The participants aged 21–30 years had significantly (*p* < 0.001) high good knowledge (24.4%) compared to other age groups. Unmarried participants had significantly (*p* = 0.01) high good knowledge (26.4%) compared to married (13.5%) participants. Participants who are university graduates demonstrated significantly (*p* = 0.03) high good knowledge (21.2%) compared to participants who got secondary school studies (9.7%). Similarly, participants who believed that the public has insufficient knowledge about epistaxis, had significantly (*p* = 0.001) lower good knowledge (15.2%) compared to their counterparts. There was no significant impact of epistaxis history on adequacy of knowledge.

**Table 2:** Association between demographic variables and level of knowledge regarding epistaxis.

Demographic variables		Level of knowledge (*N* = 452)			
		Poor (%)	Moderate (%)	Good (%)	*p* value
	Male	54 (40)	48 (35.6)	33 (24.4)	0.038*
	Female	140 (44.2)	131 (41.3)	46 (14.5)	
Age	Less than 20 years	8 (19.5)	31 (75.6)	2 (4.9)	<0.001*
	21-30 years	100 (42.4)	79 (33.5)	57 (24.4)	
	31–40 years	54 (52.9)	36 (35.3)	12 (11.8)	
	41–50 years	7 (21.2)	21 (63.6)	5 (15.2)	
	51 years and above	25 (62.5)	12 (30)	3 (7.5)	
Marital status	Unmarried	54 (34)	63 (39.6)	42 (26.4)	0.001*
	Married	131 (47.6)	107 (38.9)	37 (13.5)	
	Divorced/separated	9 (50)	9 (50)	Zero	
Education level	Illiterate	3 (42.9)	4 (57.1)	Zero	0.003*
	Intermediate education	8 (36.4)	14 (63.6)	Zero	
	Secondary school	37 (39.8)	47 (50.5)	9 (9.7)	
	University graduate	146 (44.2)	114 (34.5)	70 (21.2)	
Opinion about public has insufficient knowledge	No	3 (15)	11 (55)	6 (30)	0.001*
	Unsure	20 (46.5)	9 (20.9)	14 (32.6)	
	Yes	171 (44)	159 (40.9)	59 (15.2)	
Epistaxis during last 6 months	No	113 (44.3)	103 (40.4)	39 (15.3)	0.377
	Yes	81 (41.1)	76 (38.6)	40 (20.3)	

*p values < 0.05.

Table 3 shows the participants opinion about insufficient knowledge among public regarding epistaxis. About 86.1% of participants believe that the public does not have enough knowledge of epistaxis. A significant (*p* = 0.03) association between gender and opinion about public’s lack of knowledge was observed. We did not observe any significant impact of age, marital status, and education level on this opinion.

**Table 3:** Association between demographic variables and opinion about public knowledge regarding epistaxis.

Demographic variables		In my opinion, the public has insufficient knowledge about epistaxis (*N* = 452)			
		No (%)	Unsure (%)	Yes (%)	*p* value
Gender	Male	3 (2.2)	22 (16.3)	110 (81.5)	0.003*
	Female	17 (5.4)	21 (6.6)	279 (88)	
Age	Less than 20 years	Zero	5 (12.2)	36 (87.8)	0.096
	21–30 years	11 (4.7)	19 (8.1)	206 (87.3)	
	31–40 years	3 (2.9)	16 (15.7)	83 (81.4)	
	41–50 years	3 (9.1)	Zero	30 (90.9)	
	51 years and above	3 (7.5)	3 (7.5)	34 (85)	
Marital status	Unmarried	7 (4.4)	21 (13.2)	131 (82.4)	0.184
	Married	13 (4.7)	22 (8)	240 (87.3)	
	Divorced/separated	Zero	Zero	18 (100)	
Education level	Illiterate	Zero	Zero	7 (100)	0.442
	Intermediate education	Zero	3 (13.6)	19 (86.4)	
	Secondary school	7 (7.5)	6 (6.5)	80 (86)	
	University graduate	13 (3.9)	34 (10.3)	283 (85.8)	
	Married	13 (4.7)	22 (8)	240 (87.3)	
	Divorced/separated	Zero	Zero	18 (100)	

*p value less than 0.05.

The history of epistaxis and its association was depicted in Table 4. The epistaxis was significantly (*p* < 0.001) higher among participants aged less than 20 years (73.2%) compared to other age groups. Likewise, a significant (*p* = 0.035) impact of level of education was observed on the history of epistaxis.

**Table 4:** History of epistaxis and its association with demographic variables.

Demographic variables		History of epistaxis in the last 6 months (*N* = 452)		
		No (%)	Yes (%)	*p* value
Gender	Male	74 (54.8)	61 (45.2)	0.654
	Female	181 (57.1)	136 (42.9)	
Age	Less than 20 years	11 (26.8)	30 (73.2)	<0.001*
	21-30 years	146 (61.9)	90 (38.1)	
	31–40 years	48 (47.1)	54 (52.9)	
	41–50 years	23 (69.7)	10 (30.3)	
	51 years and above	27 (67.5)	13 (32.5)	
Marital status	Unmarried	89 (56)	70 (44)	0.986
	Married	156 (56.7)	119 (43.3)	
	Divorced/separated	10 (55.6)	8 (44.4)	
Education level	Illiterate	7 (100)	Zero	0.035*
	Intermediate education	11 (50)	11 (50)	
	Secondary school	45 (48.4)	48 (51.6)	
	University graduate	192 (58.2)	138 (41.8)	

*p value < 0.05.

## Discussion

Our study found that the average knowledge level regarding epistasis is low-to-moderate. Among the participants, 42.9%, had “Poor” knowledge score, followed by 39.6% who had “moderate” score, and 17.5% had “Good” score. These results show that most participants had poor to moderate knowledge, with a minority demonstrating a good level of knowledge. Our study reported a prevalence rate of 43.6% among participants in the last 6 months. The results of the present study corroborate with the results of a previous study by Alharethy, who reported that the age group of 41–50 years is more prone to epistaxis with a prevalence of 30.2% (27). However, another study by Al-Shehri et al. reported that 9.4% of the participants were suffering from epistaxis, which was lower than the prevalence reported in the present study (25). Because epistaxis incidence changes with age and is more prevalent in the pediatric age group, there is a wide range of incidence.

In the current study, there was a significant association between the level of knowledge and age. The highest knowledge score was among those aged between 41 and 50 years, followed by those aged less than 20 years.

Since the majority of our participants were graduates or post-graduates, it was to be expected that they had a good knowledge level of epistaxis and basic first aid, however, the average score was lower than 60%. In line with our findings, Al-Kubaisy et al. who investigated the teachers’ knowledge of managing epistaxis (23), and found that only one-third of the teachers were said to be knowledgeable about treating epistaxis, even among those who had prior exposure to epistaxis first aid knowledge. Similar findings were observed by Alshehri et al. who also found that teachers in the Alahssa region, Saudi Arabia, were not very knowledgeable on how to treat epistaxis using first aid (22). Another study by Alshehri et al. assessed school children and found that only a good proportion of them knew how to treat epistaxis using first aid (24). On the other hand, other investigations carried out in Saudi Arabia revealed a moderate-to-high degree of awareness of the first-aid treatment of epistaxis, particularly among those working in the healthcare industry, and the study findings were comparable to those of the current study. For instance, research by Alyahya et al. showed that the majority of medical students had sufficient information to be able to treat patients who have epistaxis (21). On the other hand, research among medical interns at King Fahad Armed Force Hospital revealed an insufficient understanding of epistaxis first aid (28).

It is important to raise awareness and knowledge of the general public toward epistaxis and its management and it can prevent significant complications. According to a study conducted in the US, the majority of instances of epistaxis may be effectively managed using first aid. Even though epistaxis occurs often, there was a widespread lack of understanding among the populace about first-aid care (29). A survey conducted in the United Kingdom revealed that professional healthcare practitioners as well as the general people lacked expertise and awareness about the first-aid management of epistaxis (30). Similar to the above study, a study was carried out in Glasgow to evaluate the knowledge and awareness of medical professionals working in the accident and emergency department about first-aid care of epistaxis, and the results show a lack of awareness (9).

In a previous research, participants with a history of epistaxis were asked about their first-aid management expertise and advice received, the study revealed that epistaxis patients who were treated by general practitioners and other non-ENT medical or nursing professionals could not recall receiving first aid guidance. In contrast, patients who had previously been seen by the ENT staff scored full points in every aspect (19). Another study was carried out in Germany to evaluate the effectiveness of ice packing as a first-aid management strategy, no discernible differences were seen between the blood vessels inside the nasal mucosa before and after the administration of ice packs to the neck regions (31). Additionally, a study carried out in Ireland evaluated the impact of epistaxis on the lives of parents and their affected children, and it was recommended to raise educational and public knowledge levels on the condition’s first care (4). Furthermore, an Australian instance with internal carotid artery rupture and epistaxis was recorded. The study’s findings indicated that to prevent fatalities in such situations, more awareness and vigorous first-aid care are required (32).

Increased blood loss, unwarranted hospitalizations, and even fatalities will occur as a result of improper first-aid care of epistaxis. In a study conducted in Kenya, Mugwe et al. noted that YouTube was a popular source for learning how to treat epistaxis, but they also voiced worry about the credibility of such videos (26). Nasal pressure is a crucial strategy for stopping bleeding during an acute bout of epistaxis.

### Strengths and limitations

The current study has a number of strengths worth mentioning. Firstly, the study has important implications for healthcare providers and policymakers in Saudi Arabia. The findings of the study can be used to develop targeted interventions and educational programs that improve the knowledge and awareness of epistaxis among the general public, which can ultimately lead to better health outcomes. Secondly, the study employed an optimum sample size, making it possible to minimize the danger of reporting false-positive or false-negative results. Our research has some limitations as well. Since all of our participants were Saudi nationals and adults, our findings do not reflect the knowledge and awareness of minors and non-Saudi population residing in Saudi Arabia. Selection bias in online recruitment cannot be ruled out, since there may be inherent biases in the individuals who are exposed to and choose to respond to the questionnaire. This can result in a sample that is not representative of the broader population of interest. Online questionnaires may be prone to social desirability bias, where respondents may be inclined to provide answers that they perceive as socially acceptable or desirable rather than their true opinions or behaviors.

## Conclusion

Our study found a non-satisfactory, low-to-moderate knowledge level of the Saudi general public toward epistaxis. We recommend emphasis to improve the public awareness and educating them about first aid as it when managed properly, can reduce significant complications. As a result, we strongly suggest that first aid camps and seminars on the management of epistaxis be held in order to provide the general people with the appropriate education they need to appropriately manage the condition.

## Data availability statement

The original contributions presented in the study are included in the article/supplementary material, further inquiries can be directed to the corresponding author.

## Ethics statement

The studies involving humans were approved by Institutional Review Board of Najran University. The studies were conducted in accordance with the local legislation and institutional requirements. The participants provided their written informed consent to participate in this study.

## Author contributions

AA: Writing – original draft, Conceptualization.

## Footnotes

^1^https://worldpopulationreview.com/world-cities/riyadh-population

^2^http://www.raosoft.com/samplesize.html, accessed on 30 May 2022.
